# p16 overexpression and 9p21 deletion are linked to unfavorable tumor phenotype in breast cancer

**DOI:** 10.18632/oncotarget.13227

**Published:** 2016-11-09

**Authors:** Patrick Lebok, Magdalena Roming, Martina Kluth, Christina Koop, Cansu Özden, Berivan Taskin, Khakan Hussein, Annette Lebeau, Isabell Witzel, Linn Wölber, Stefan Geist, Peter Paluchowski, Christian Wilke, Uwe Heilenkötter, Volkmar Müller, Barbara Schmalfeldt, Ronald Simon, Guido Sauter, Luigi Terracciano, Rainer Horst Krech, Albert von der Assen, Eike Burandt

**Affiliations:** ^1^ Institute of Pathology, University Medical Center Hamburg-Eppendorf, Hamburg, Germany; ^2^ Department of Gynecology, University Medical Center Hamburg-Eppendorf, Hamburg, Germany; ^3^ Department of Gynecology, Regio Clinic Pinneberg, Pinneberg, Germany; ^4^ Department of Gynecology, Regio Clinic Elmshorn, Elmshorn, Germany; ^5^ Department of Gynecology, Clinical Centre Itzehoe, Itzehoe, Germany; ^6^ Department of Pathology, Basel University Clinics, Basel, Switzerland; ^7^ Institute of Pathology, Clinical Centre Osnabrück, Osnabrück, Germany; ^8^ Breast Centre Osnabrück, Osnabrück, Germany

**Keywords:** breast cancer, 9p21 deletion, TMA, p16 expression, CDKN2A

## Abstract

Overexpression of the p16 tumor suppressor, but also deletion of its gene locus 9p21, is linked to unfavorable tumor phenotype and poor prognosis in breast cancer. To better understand these contradictory observations, and to clarify the prognostic impact of p16 expression and 9p21 deletion, a tissue microarray (TMA) with 2,197 breast cancers was analyzed by fluorescence *in-situ* hybridization and immunohistochemistry (FISH) for 9p21 deletion and p16 expression. p16 immunostaining was weak in 25.6%, moderate in 7.1%, and strong in 12.7% of 1,684 evaluable cancers. Strong p16 staining was linked to advanced tumor stage (*p* = 0.0003), high-grade (*p* < 0.0001), high tumor cell proliferation (*p* < 0.0001), negative hormone receptor (ER/PR) status (*p* < 0.0001 each), and shorter overall survival (*p* = 0.0038). 9p21 deletion was found in 15.3% of 1,089 analyzable breast cancers, including 1.7% homozygous and 13.6% heterozygous deletions. 9p21 deletion was linked to adverse tumor features, including high-grade (*p* < 0.0001) and nodal positive cancers (*p* = 0.0063), high cell proliferation (*p* < 0.0001), negative hormone receptor (ER/PR) status (*p* ≤ 0.0006), and HER2 amplification (*p* = 0.0078). Patient outcome was worse in 9p21 deleted than in undeleted cancers (*p* = 0.0720). p16 expression was absent in cancers harboring homozygous 9p21 deletions, but no difference in p16 expression was found between cancers with (59.2% p16 positive) and without heterozygous 9p21 deletion (51.3% p16 positive, *p* = 0.0256). In summary, p16 expression is unrelated to partial 9p21 deletion, but both alterations are linked to aggressive breast cancer phenotype. High-level p16 expression is a strong predictor of unfavorable disease course in breast cancer.

## INTRODUCTION

Breast cancer is the most common malignancy detected in women [1]. Surgical removal of the cancer followed by adjuvant therapy represents the standard of care. Accurate prediction of recurrence risk is of vital importance for tailoring adjuvant therapy for individual breast cancer patients. Conventional pathological parameters, such as histological grade, tumor size, and presence of lymph node metastasis, are not accurate enough to select subsets of patients who are at sufficiently low risk of recurrence to be spared from extensive adjuvant therapy without compromising prognosis. Accumulating evidence exists that additional molecular testing can help to better select patients who would benefit most from adjuvant therapy, at the same time sparing those who would derive little or no advantage from treatment [2–4].

The p16 tumor suppressor, encoded by the *CDKN2A* gene at 9p21, has been discussed as a prognostic factor in breast cancer for more than a decade. p16 inhibits cyclin-dependent kinases (CDKs) 4 and 6 at the G1 to S phase transition, thus preventing phosphorylation of the retinoblastoma (RB1) protein. Insufficiently phosphorylated RB1 leads to sequestered E2F in an incompetent RB1/E2F complex, preventing E2F from triggering cell cycle progression (reviewed in [5]). p16 plays a pivotal role in various tumor types including cancers of colon, skin, and gallbladder (reviewed in [6]). In breast cancer, studies on 10–314 patients suggested a role of p16 overexpression in tumor progression [7–10], metastasis [10], and clinical outcome [9, 10]. It is, thus, remarkable that deletion of the chromosomal region 9p21, potentially leading to reduced p16 expression, belongs to the most frequent deletions in breast cancers occurring in 11% to 65% [11–17]. Two of these studies with 39 and 166 cancers even described a link between 9p21 deletions and unfavorable tumor phenotype [15, 16].

To evaluate the potential role of both p16 expression and 9p21 deletion as prognostic features we investigated a cohort of more than 2,100 breast cancers employing immunohistochemistry (IHC) and fluorescence *in-situ* hybridization (FISH).

## RESULTS

### Prevalence of p16 expression and 9p21 deletion in breast cancer

**Figure 1:**
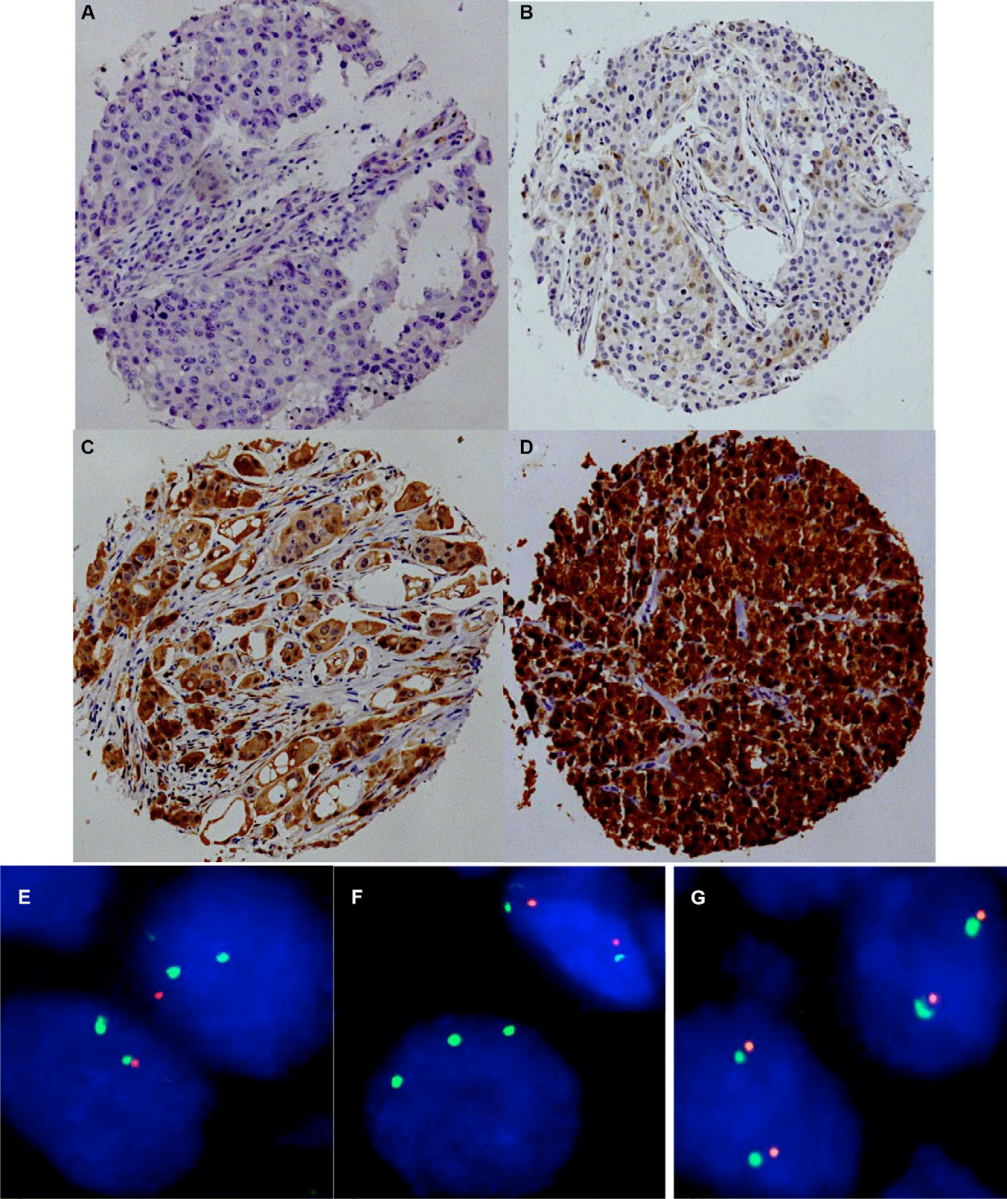
Representative images of p16 immunostaining with (A) negative, (B) weak, (C) moderate, and (D) strong staining and examples of FISH findings using the 9p21 deletion probe (**E**) Heterozygous deletion as indicated by the lack of one orange 9p21 signal and two green centromere 9 signals, (**F**) Homozygous deletion as indicated by the complete lack of orange 9p21 signals in the tumor cell, (**G**) Normal 9p21 copy numbers as indicated by two orange 9p21 signals and two green centromere 9 signals.

To estimate the baseline level of p16 expression we analyzed 20 normal breast tissues. In normal breast epithelium p16 immunostaining was usually negative or limited to a small fraction of cells (< 5%) and appeared less intense (mostly weak to moderate) as compared to p16 positive cancer cells. P16 immunostaining was observed in 747 of 1,684 (44.4%) analyzable cancers. If present, p16 staining was typically found in all tumor cells, and was considered weak in 25.6%, moderate in 7.1% and strong in 12.7% of cancers. Representative images of immunostainings are shown in Figure 1A–1D. 9p21 was successfully analyzed by FISH in 1,089 (49.6%) arrayed cancer samples. 9p21 deletions were found in 167 (15.3%) interpretable breast cancers, including 13.6% heterozygous and 1.7% homozygous deletions (Figure 1E–1G).

### Association of p16 expression and 9p21 deletion to breast cancer phenotype

Strong p16 expression was tightly linked to adverse tumor features, including histopathological grade (*p* < 0.0001), advanced tumor stage (*p* = 0.0003), and hormone receptor (ER/PR) negativity (*p* < 0.0001 each) in all breast cancers and in the largest subset of cancers of No Special Type (NST; *p* ≤ 0.0010). Also 9p21 deletion was significantly associated with unfavorable tumor features, including histological grade (*p* < 0.0001), presence of lymph node metastases (*p* = 0.0063), ER/PR negativity (*p* < 0.0001 for ER and *p* = 0.0006 for PR), and amplifications of *HER2* (*p* = 0.0078) in all breast cancers. These associations held also true in the subset of NST cancers *p* < 0.05). All results are summarized in Table 1.

**Table 1:** Clinico-pathological association of 9p21 deletion (FISH) and p16 expression (IHC)

		9p21 FISH results			p16 IHC results				
		analyzable (n)	deletion (%)	*p*-value	analyzable (n)	weak (%)	moderate (%)	strong (%)	*p*-value
All cancers		1089	15.34		1684	25.59	7.07	12.71	
Histology	No special type	809	15.82		1144	28.41	7.17	13.37	
	Lobular carcinoma	110	10.91		210	18.57	4.76	5.71	
	Cribriform carcinoma	35	11.43		49	42.86	8.16	0.00	
	Medullary carcinoma	32	21.88		44	2.27	9.09	54.55	
	Tubular carcinoma	23	13.04		38	15.79	5.26	0.00	
	Papillary carcinoma	16	18.75		22	13.64	27.27	31.82	
	Mucinous carcinoma	30	3.33		50	24.00	6.00	8.00	
	Other rare types^*^	25	24.00		83	18.10	8.40	14.50	
pT stage	pT1	365	12.88	0.1565	547	27.06	5.67	8.59	0.0003
	pT2	528	16.29	^**^0.1056	787	26.43	8.51	15.37	^**^0.0009
	pT3	53	24.53		96	19.79	9.38	20.83	
	pT4	137	14.60		198	25.25	4.55	11.62	
BRE grade	Grade 1	248	6.05	<0.0001	396	27.27	6.57	3.28	< 0.0001
	Grade 2	387	12.66	^**^< 0.0001	598	27.09	6.35	6.69	^**^< 0.0001
	Grade 3	368	23.37		510	23.92	8.63	29.61	
Nodal stage	pN0	467	13.06	0.0063	678	27.14	7.96	12.83	0.3911
	pN1	387	18.09	^**^0.0324	592	25.51	6.25	14.02	^**^0.8723
	pN2	60	28.33		96	26.04	10.42	18.75	
ER status	Negative	253	24.11	< 0.0001	369	15.72	8.67	40.92	< 0.0001
	Positive	801	12.48	^**^0.0037	1180	29.66	7.03	4.92	^**^< 0.0001
PR status	Negative	640	17.97	0.0006	980	23.98	7.14	17.14	< 0.0001
	Positive	366	10.11	^**^0.0099	516	31.78	7.75	6.01	^**^< 0.0001
HER2 status	no amplification	776	14.30	0.0078	1074	26.26	7.17	12.57	0.0829
	amplification	176	22.73	^**^0.0451	233	26.18	9.01	18.03	^**^0.4391

* including adenoid-cystic carcinoma, apocrine carcinoma, atyp medullary carcinoma, carcinosarcoma, clear cell carcinoma, histiocytic carcinoma, lipid rich carcinoma, lipid rich or histiocytoic carcinoma, metaplastic carcinoma, neuroendocrine carcinoma, signet ring carcinoma, and small cell carcinoma.

** in subgroup of cancers of No Special Type

### Association of p16 expression and 9p21 deletion with cell proliferation

Data on the tumor cell proliferation, as determined by immunohistochemical analysis of the Ki67 antigen, were available from a previous study using the same TMA [18]. Strong p16 staining as well as 9p21 deletions were tightly associated with a high Ki67 labeling index (LI) if all cancers were jointly analyzed (*p* < 0.0001 each). These associations were independent from the histological grade because they held also true in the subsets of cancers with identical grade. All results are summarized in Table 2.

**Table 2:** Association between 9p21 deletion or p16 expression and Ki67-labeling index

	9p21 FISH					p16 IHC				
		normal	deletion			negative	weak	moderate	strong	
	analyzable (n)	Ki67LI	Ki67LI	*p*-value	analyzable (n)	Ki67LI	Ki67LI	Ki67LI	Ki67LI	*p*-value
**All cancers**	947	28.70 ±0.52	36.37 ±1.23	*p* < 0.0001	1425	24.63 ±0.50	27.38 ±0.72	29.16 ±1.33	41.48 ±0.99	*p* <0.0001
**Grad 1**	211	19.42 ± 0.72	25.36 ± 3.07	*p* = 0.0610	337	18.26 ± 0.69	17.47 ± 1.08	20.00 ± 2.12	24.27 ± 3.07	*p* = 0.1729
**Grad 2**	343	25.63 ± 0.66	32.58 ± 1.69	*p* = 0.0001	535	23.87 ± 0.67	25.80 ± 1.00	25.97 ± 1.96	29.00 ± 1.96	*p* = 0.0494
**Grad 3**	319	40.20 ± 0.96	40.23 ± 1.73	*p* = 0.9907	443	34.25 ± 1.12	36.84 ± 1.34	35.23 ± 2.21	46.00 ± 1.19	*p* < 0.0001

### Prognostic relevance of p16 expression and 9p21 deletion

**Figure 2:**
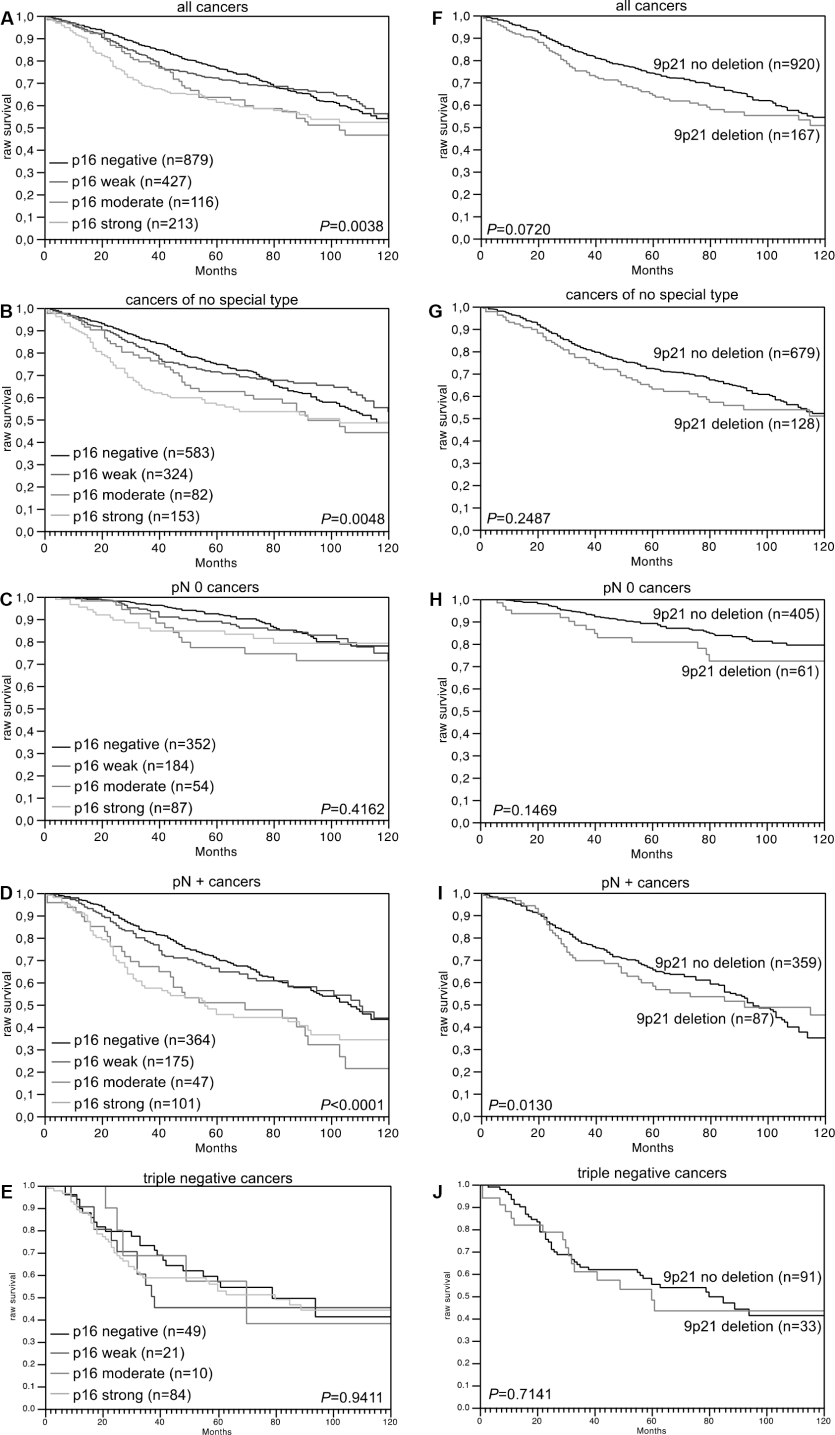
Association between p16 expression and raw survival in (A) all cancers, (B) no special type cancers, (C) nodal negative cancers, (D) nodal positive cancers, and (E) triple negative cancers Association between 9p21 deletion and raw survival in (**F**) all cancers, (**G**) no special type cancers, (**H**) nodal negative cancers, (**I**) nodal positive cancers, and (**J**) triple negative cancers.

Data on raw survival were available from 1,635 cancers with interpretable p16 IHC results and from 1,087 cancers with interpretable 9p21 FISH results. Strong p16 staining was linked to shortened overall survival if all cancers were jointly analyzed (*p* = 0.0038, Figure 2A), as well as in the subsets of NST cancers (*p* = 0.0048, Figure 2B), and in the subset of cancers with nodal metastases (*p* < 0.0001, Figure 2D). No association was found between p16 expression and overall survival in the subset of triple negative cancers (*p* = 0.9411, Figure 2E). No unequivocal association was found between 9p21 deletion and overall survival, neither in all cancers (*p* = 0.0720) nor in subsets of NST (*p* = 0.2478), nodal negative (*p* = 0.1469), nodal positive (*p* = 0.0130), or triple negative cancers (*p* = 0.7141, Figure 2F–2J). In a multivariate analysis including KI67LI, hormone receptor status, HER2 status, pT stage, BRE grade and nodal stage, p16 expression predicted overall survival independently from these parameters in 800 analyzable cancers (*p* = 0.0304, Table 3).

**Table 3:** Multivariate analysis (Cox regression) including pathological and molecular parameters in addition to p16 expression in all cancers

Parameter	RR	95%CI	*p*-Value
**KI67LI**	1.3	0.6–2.7	0.4918
**ER status**			
positive vs negative	0.8	0.6–1.2	0.3189
**PR status**			
positive vs negative	0.6	0.4–0.8	0.0005
**HER2 status**			
positive vs negative	1.1	0.8–1.5	0.6347
**pT stage**			
2 vs 1	1.2	0.8–1.6	0.0007
3 vs 2	1.2	0.7–1.7	
4 vs 3	1.7	1.0–2.8	
**pN stage**			
N+ vs N0	2.9	2.2–3.9	< 0.0001
**BRE grade**			
2 vs 1	1.3	0.8–1.9	< 0.0001
3 vs 2	2.3	1.7–3.1	
**p16 IHC**			
weak vs negative	1.1	0.8–1.5	0.0145
moderate vs weak	1.5	0.9–2.2	
strong vs moderate	0.5	0.3–0.7	

### Relationship between p16 expression and 9p21 deletion

**Figure 3:**
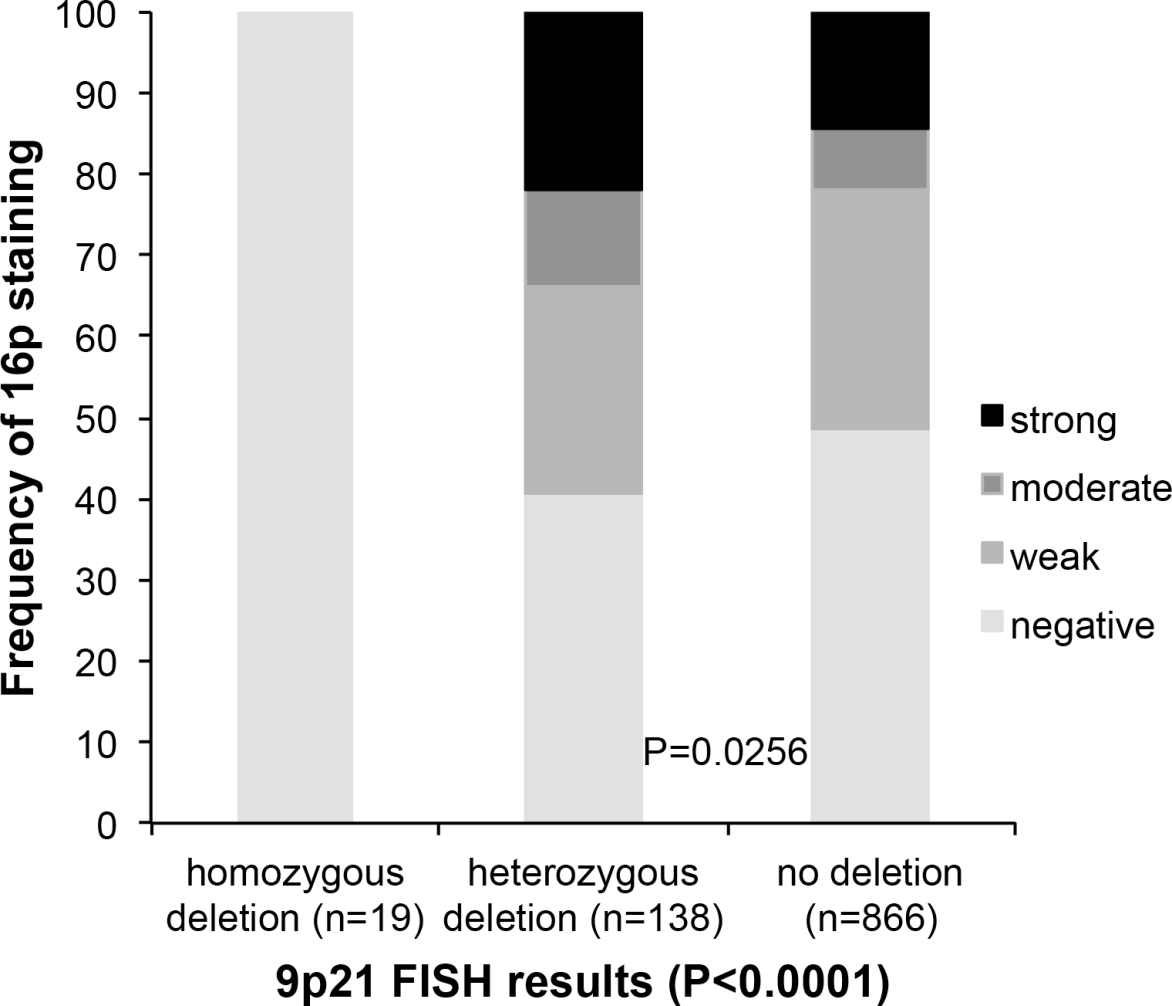
Association between 9p21 deletion (FISH) and p16 expression (IHC)

In all (100%) of 19 cancers with homozygous 9p21 deletion p16 immunostaining was completely absent. No significant difference of p16 expression was found between 138 cancers with heterozygous 9p21 deletions and 866 cancers without 9p21 deletion. p16 expression was even slightly higher in deleted than in undeleted cancers (*p* = 0.0256, Figure 3).

## DISCUSSION

The results of our study on 2,100 breast cancers identify that p16 overexpression and 9p21 deletion are largely independent of each other while both features are linked to aggressive breast cancer phenotype.

Our IHC analysis shows that p16 is up regulated in a fraction of tumors as compared to normal breast epithelium. Under the selected staining conditions, positive p16 immunostaining was found in about 50% of breast cancers, including more than 10% of cancers with high intensity p16 staining. Earlier immunohistochemistry studies using TMAs and large sections examining 20–119 breast cancers reported frequencies of p16 positivity ranging from 56% to 89% (TMA) and 21% to 51% (large sections) [8, 9, 19–23]. Such discrepancies are common if a protein is analyzed by immunohistochemistry in a large number of studies. They are typically attributable to the usage of different antibodies, laboratory protocols, and scoring criteria. However, the comparably high fraction of p16 positive cases in our study using a single 0.6 mm TMA spot per cancer and earlier studies on conventional large sections argues against substantially heterogeneity of p16 expression in breast cancer.

Despite of its well-known role as a tumor suppressor gene, p16 overexpression - but not expression loss - was linked to adverse tumor parameters, including advanced stage, high grade and shorter survival independently from known prognosticators, including pT stage, nodal stage, BRE grade, hormone receptor state, HER2 and cell proliferation. Most of these associations are in line with earlier IHC studies examining 10, 60, 82 and 314 breast tumors that had already suggested associations between p16 overexpression and adverse features of breast cancer such as high grade [7–10], nodal stage [10] and poor patient prognosis [9, 10]. That p16 expression lacked prognostic relevance in the subset of triple negative cancers underscores the particularly poor prognosis of these cancers [24].

Finding a close association between p16 positivity and accelerated cell proliferation fits well to the specific role of p16 for cell cycle regulation. p16 is up regulated in G1 phase of each cell cycle and has an exceptionally long half-life time as compared to other cell cycle regulators (reviewed in [5, 25]). In slowly proliferating cells with a doubling time greater than the p16 dismantling period, it can, thus, be expected that p16 is completely cleared from the cell between two mitoses. Accordingly, we observed scattered p16 staining only in a low fraction of normal breast epithelial cells, which corresponds to the fraction of proliferating cells (< 3%). In contrast, rapidly proliferating cancers showed uniformly strong accumulation of p16 in virtually all cells, indicating that p16 clearance was not accomplished between two mitoses.

Another aim of our study was to clarify the relationship between 9p deletion and p16 expression, both of which have been suggested as markers for breast cancer progression. As expected, cancers harboring complete (i.e., homozygous) 9p deletion lacked p16 expression, which indirectly validates our experimental approaches both for FISH and IHC. That no difference of p16 expression was seen between cancers with and without heterozygous 9p deletion demonstrates that breast cancer cells are able to fully compensate for loss of one p16 (*CDKN2A*) allele, either by increased transcriptional activation of the remaining allele or by increased stabilization of p16 protein or mRNA (reviewed in [5, 25]). Our findings are in line with published data from the TCGA project showing no significant differences in p16 expression between cancers with partial (heterozygous) deletion and normal copy numbers (http://www.cbioportal.org [26, 27]). These results, therefore, demonstrate that heterozygous 9p deletion is not a relevant mechanism for altering p16 expression in breast cancer. We conclude from these observations that p16 may not represent the main target gene of 9p deletions in breast cancer. Complete p16 inactivation occurs in only 1.5% of 9p deleted cancers by homozygous deletion and inactivating p16 mutations – that might accompany some of the 9p deletions – occur in only 0.5 to 7% of breast cancers [12, 28–31]).

Finding 9p deletions in 15% of cancers fits well to earlier work reporting deletions of 9p in 6–25% by classical or array CGH in cohorts of 39–98 analyzed breast cancers [12, 15, 32, 33]. Higher rates of 9p deletions were only found in studies selecting for metastatic breast cancers (41% of 34) [34] or studies employing less quantitative loss of heterozygosity (LOH, 11–65%) analyses on 12–171 cases [11, 13, 14, 17]. These assays are influenced by ploidy changes and admixture of normal cells, which inevitably affect the assay specificity and sensitivity. In contrast, FISH allows for precise gene copy number determination in individual cells, rendering it independent from the purity of cancer tissues or presence of aneusomy. FISH is thus considered the gold standard for gene copy number analysis.

9p deletions were linked to features of aggressive breast cancer such as high-grade and a strong trend towards reduced survival was found. Studies using classical CGH have demonstrated that 9p deletions typically comprise large portions of 9p or even the entire chromosome arm [32, 33]. The example of p16 shows that not all effected genes must necessarily become down-regulated in 9p deleted cancers, but it seems likely that many genes will be. These might include genes with tumor suppressive properties, such as *CDKN2B* (9p21) [35], *SH3GL2* (9p22) [36], *PTPRD* (9p23) [37], and *DOCK8* (9p24) [38]. Such genes may, either alone or in concert contribute to tumor progression when affected by 9p deletion. A cooperative effect of genes hit by large deletions has been demonstrated for large 8p deletions in liver cancer [39].

In conclusion, the results of our study show that strong p16 overexpression occurs in 10% of breast cancers and is linked to a fraction of aggressive and rapidly proliferating breast cancers with poor prognosis.

## MATERIALS AND METHODS

### Breast cancer tissue microarray (TMA)

A pre-existing tissue microarray (TMA) was used for this study [18]. The TMA contained 2,197 human breast cancer samples from paraffin-embedded tissue specimens fixed in 4% neutral buffered formalin. From each patient one 0.6 mm core was taken from a representative cancer tissue block. All tissues were distributed among 6 TMA blocks, each containing 263 to 522 tumor samples. Consecutive breast cancer samples collected between 1984 and 2000 were used for this study. The median patient's age was 63 (range 26–101) years. The use of the specimens and data for research purposes was approved by the Ethics Committee of the Basel University Hospital. Survival data were either obtained from the cancer registry of Basel or collected from the patients attending physicians. Raw survival data were available from 1,982 patients (713 patients with and 1,508 without event). The mean follow-up time was 63 months (range 1–176 months). Tumor size and nodal status were obtained from the primary pathology reports. All slides from the tumors were reviewed by specialized pathologists to define the histologic grade according to Elston and Ellis [40] and the histologic tumor tumor type. Four μm sections of the TMA blocks were transferred to an adhesive coated slide system (Instrumedics Inc., Hackensack, New Jersey) for FISH and IHC analysis. Molecular data used in this study were available from previously published studies. These included amplification data obtained by FISH for *HER2* amplification as well as IHC data on estrogen receptor (ER) and progesterone receptor (PR) expression as well as Ki67 labeling index (Ki67 LI) [18, 41].

### Fluorescence *in-situ* hybridization

Four micrometer TMA sections were used for FISH. For proteolytic slide pretreatment, a commercial kit was used (paraffin pretreatment reagent kit; Abbott, Wiesbaden, Germany). TMA sections were deparaffinized, air-dried, and dehydrated in 70%, 85%, and 100% ethanol, followed by denaturation for 5 min at 74°C in 70% formamid 2x SSC solution. The commercial Vysis *CDKN2A* / CEP 9 FISH probe kit (#04N61–020; Abbott, Wiesbaden, Germany) was used for detection of the 9p21 status. Hybridization was performed overnight at 37°C in a humidified chamber. Slides were subsequently washed and counterstained with 0.2 μmol/L 4′-6-diamidino-2-phenylindole in antifade solution. Stained slides were manually interpreted with an epifluorescence microscope by expert scientists (MK, CÖ, BT, KH and MR). The predominant FISH signal numbers were recorded in each tissue spot, and a consensus result was generated in case of unequivocal findings. Presence of fewer *CDKN2A* signals than centromere 9 probe signals in at least 60% tumor nuclei was considered a heterozygous deletion. Complete absence of *CDKN2A* signals in all tumor cells, but presence of centromere 9 and *CDKN2A* signals in adjacent normal cells, was considered a homozygous deletion. Tissue spots lacking any detectable *CDKN2A* signals in all (tumor and normal cells) or lack of any normal cells as an internal control for successful hybridization of the *CDKN2A* probe were excluded from analysis. These thresholds were based on our previous study analyzing *PTEN* deletions on a prostate cancer TMA where our approach resulted in a 100% concordance with array comparative genomic hybridization (CGH) data [42].

### Immunohistochemical analysis

Freshly cut TMA sections were immunostained on one day and in one experiment. Slides were deparaffinized, rehydrated, washed in DAKO buffer (K8002) and transferred to a DAKO Link 48 autostainer device. The immunohistochemical staining of p16 was performed with the commercially available CINtec p16 Histology Kit (Cat.# 725–4713, Ventana Medical Systems Inc., Arizona, USA) according to the manufacturer's instructions. p16 staining was typically nuclear and cytoplasmic. The staining intensity (0, 1+, 2+, and 3+) and the fraction of positive tumor cells were separately recorded for each tissue spot by a pathologist (PL). A final score was then built from these 2 parameters according to the following score as previously described [43, 44]. Negative scores had complete absence of staining, weak scores had staining intensity of 1+ in ≤ 70% of tumor cells or staining intensity of 2+ in ≤ 30% of tumor cells; moderate scores had staining intensity of 1+ in > 70% of tumor cells, staining intensity of 2+ in > 30% but in ≤ 70% of tumor cells or staining intensity of 3+ in ≤ 30% of tumor cells; strong scores had staining intensity of 2+ in > 70% of tumor cells or staining intensity of 3+ in > 30% of tumor cells.

### Statistics

Statistical calculations were performed with JMP 9 software (SAS Institute Inc., NC, USA). Contingency table analysis and Chi square test were used to study the relationship between IHC and FISH results and clinicopathological variables. Kaplan–Meier plots were used to estimate overall survival and the statistical significance was determined by the log rank test. The log-Rank test was applied to test the significance of differences between stratified survival functions. Cox proportional hazards regression analysis was performed to test the statistical independence and significance between pathological and molecular variables.
